# Development and Validation of the Coeliac Disease Food Attitudes and Behaviours Scale

**DOI:** 10.1155/2018/6930269

**Published:** 2018-08-19

**Authors:** Rose-Marie Satherley, Ruth Howard, Suzanne Higgs

**Affiliations:** ^1^School of Populations Sciences and Health Services Research, Guy's Campus, King's College London, 5th Floor Addison House, London SE1 1UL, UK; ^2^School of Psychology, University of Birmingham, 52 Pritchatts Road, Edgbaston, Birmingham B15 2SB, UK

## Abstract

**Objectives:**

Previous studies on coeliac disease suggest that attitudes towards the gluten-free diet may contribute to the development of disordered eating. This study describes the development and validation of the Coeliac Disease Food Attitudes and Behaviours scale (CD-FAB) to measure these behaviours in coeliac disease.

**Research Methods and Procedures:**

Focus groups were used to develop 33 potential questionnaire items. These items were reviewed by service users and then distributed online to 157 adults with coeliac disease. Items were removed based on ceiling/floor effects, high interitem correlations (>0.7) and factor analysis. 11 items were retained. Exploratory factor analysis was then conducted. The psychometric properties of the final version of the CD-FAB were assessed via using an online platform.

**Results:**

The CD-FAB had 11 items distributed across one factor assessing attitudes and behaviours towards food. These factors explained 44.1% of the variance in responding. The CD-FAB and its subscales had high internal consistency (Cronbach's alpha > 0.7) and psychometric validation indicated good convergent and discriminant validity. High scores on the CD-FAB are associated with psychological distress and an impaired quality of life.

**Conclusions:**

The CD-FAB is a reliable and valid measure of food attitudes and behaviours in coeliac disease. As a new disease-specific instrument, it may be a useful tool for evaluating food concerns in individuals with coeliac disease in a clinical setting and for further exploring the development of disordered eating patterns in coeliac disease. Further research is required to assess the full potential of the CD-FAB.

## 1. Introduction

Coeliac disease (CD) is managed by a strict, life-long gluten-free diet (GFD), which leads to improvement in the majority of CD-related symptoms [1]. However, following the GFD may harm some individuals' relationship with food, increasing the risk of disordered eating [2]. Disordered eating describes a spectrum of eating behaviours, ranging from clinical eating disorders, including anorexia and bulimia nervosa [3], to skipping meals and restricting certain foods [4].

A two-pathway model of disordered eating has been used to understand disordered eating attitudes and behaviours in CD [2]. The first pathway describes those who experience distress around weight change associated with diagnosis that triggers disordered eating attitudes and behaviours. The second pathway describes a hypervigilance around food that triggers disordered eating attitudes and behaviours. Case studies of comorbid CD and disordered eating, although limited by small sample sizes, are concordant with systematic studies suggesting that 22–29% of individuals with CD score above clinical cut-offs for disordered eating [5–7]. However, evidence for clinically diagnosed eating disorders is mixed. Evidence from Sweden and the UK had demonstrated an increased risk of anorexia nervosa in CD [8, 9], whereas Babio et al. [10] found no greater risk for eating disorder in individuals with CD when compared to healthy controls.

Following the GFD requires vigilance around food and knowledge of food preparation and so current disordered eating measures may incorrectly classify behaviours essential for the management of CD as disordered. The binge eating scale (BES) [11] and the Eating Attitudes Test (EAT-26) [12] have been used to identify disordered eating CD [6]. However, some individuals with CD who score below the clinical cut-off on these measures describe apparent disordered eating attitudes and behaviours, with a cognitive focus on fears about cross-contamination and food safety [13]. Although control around food is essential for those following a GFD, these beliefs about food may result in disordered eating attitudes and behaviours [13]. To better understand how these beliefs develop from adaptive coping mechanisms to disordered attitudes and behaviours, they must be measureable in CD.

This article reports the development of a CD Food Attitudes and Behaviours Scale (CD-FAB) to identify disordered eating attitudes and behaviours resulting from beliefs around cross-contamination and food safety, including the psychometric properties comprising subscale structure, reliability and validity, and psychosocial correlates.

## 2. Methods

**Figure 1:**
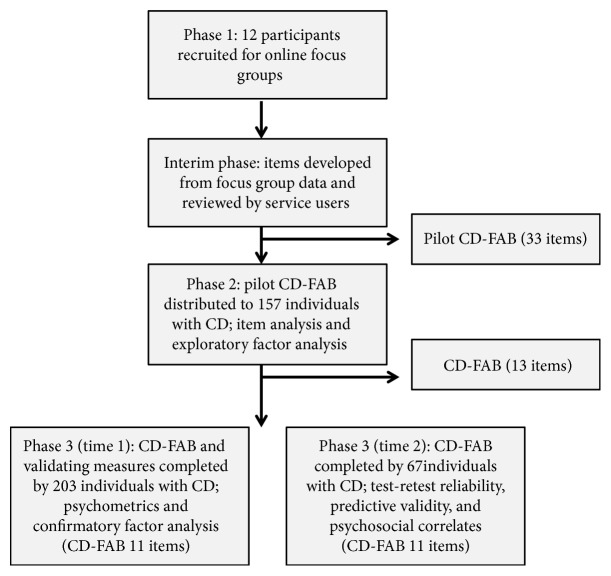
Flow chart of the CD-FAB development and validation process.

A mixed methods approach using three studies was used to develop the CD-FAB (Figure 1).

## 3. Study 1: Item Generation

Online focus groups, moderated by the first author, were used to generate CD-FAB items. Individuals with a self-reported, biopsy-confirmed diagnosis of CD (18–69 years) were recruited from online forums. Individuals reporting CD diagnosis via blood test, physician report, or self-report, without intestinal biopsy, were excluded from the analyses. Interested individuals emailed the first author to complete a screening questionnaire and to confirm focus group attendance. Verbal consent, over the phone, and online consent was provided before attending the focus group. Participants experiencing other dietary-controlled conditions (e.g., cystic fibrosis and diabetes mellitus) or food allergies were excluded.

Eight open-ended questions were designed to answer the key questions of the study: (1) the construct of food attitudes in CD and (2) the everyday interactions with food in CD. The five stages in the two-pathway model of disordered eating in gastrointestinal disease (diagnosis of CD, adaptation to diagnosis, illness beliefs, dietary management, and eating patterns) were used to frame questions [2]. Closure of the focus group was based on data saturation, by repeatedly comparing data across participants, which occurred when no new information was obtained from the focus group.

### 3.1. Data Analysis

Thematic analysis identified key themes related to food attitudes, concerns, and eating behaviours [14]. The first author (experienced in qualitative analysis and under the supervision of experienced academics) read through the transcripts noting initial thoughts; transcripts were reread allowing data immersion. Subsequently, the coding phase began. Codes identified characteristics related to food attitudes, concerns, and eating behaviours; similar codes were grouped, creating themes. Emerging themes were used to develop items for the CD-FAB. Each item was transformed into a 7-point Likert scale (strongly agree to strongly disagree). Questions were phrased so that higher scores indicated greater food concerns; four items were reverse scored to minimise response bias. Individuals from the focus group rated the 33 pilot items on clarity, adequacy, and relevance to the focus group discussion. Three service users with CD, recruited via email from the Birmingham Coeliac UK committee who did not take part in the focus groups, commented on the clarity, adequacy, and relevance of the questions. These questions were rated on a 5-point scale (1: strongly agree to 5: strongly disagree); those items that consistently scored low were removed.

## 4. Results of Item Generation

Twelve individuals took part in the focus group (10 females, mean age = 29.1 years, SD = 8.16; mean time since diagnosis = 6.2 years, SD = 4.69). Three had a CD diagnosis for over 10 years (see Table 1).

### 4.1. Thematic Analysis

Four themes were identified: *handling of food*, *trust*, *risk-taking*, and *food safety. Handling of food* refers to feelings around gluten-containing products, including preparing gluten-containing food for others, having gluten in the home, and touching gluten-containing foods. Some participants said that they would prepare gluten-containing foods for others and had no concerns being around gluten, as long as they did not consume gluten. Other participants described a fear around food that was attributed to their need to be vigilant about food content; gluten was not allowed in the home and feelings of anxiety increased when they were in close proximity to gluten. “*I get concerned in supermarkets when the gluten-free bread is next to the normal bread. I know they're all wrapped up but it scares me” (Ashley).*

The second theme, *trust*, described the need for control during food preparation, especially where others were involved. Concerns stemmed from the belief that others may not be vigilant around cross-contamination. To reduce concerns around eating food prepared by others, trust in the individual preparing food was needed. “*I don't let him prepare my food. I like to be in control of my food, I can't trust others to do it” Charlie).* Clem described the impact of trust on the ability to eat outside the home “*Going out for a meal I always have to double check. If they (the restaurant) sound unsure I won't go. I need to trust them” (Clem).*

*Risk-taking* reflects the ability to consume foods in new environments. Twelve participants indicated that an element of risk was necessary to live a normal life. A lack of risk-taking led to isolation from events involving food. “*I have to take small risks if I want to have a normal life!” (Jamie).*

*Food safety* describes the eating strategies employed to manage food concerns. Although most participants were willing to try new gluten-free foods, some viewed food as the enemy. These individuals experienced anxiety around food and felt safer not eating. A limited range of food was consumed, or long periods of food restriction to promote safety and prevent gastrointestinal symptoms were reported. “*I cope with my fear of getting glutened by not eating.” (Ashley).* These attitudes were related to the participant's ability to recall their symptoms and adverse food experiences prior to diagnosis. “*I don't go to restaurants. They remind me of being ill. I didn't like that, so I don't eat much” (Alex).*

### 4.2. Item Development

The identified themes were used to generate 33 items for the CD-FAB. After the review, 13 items were reworded and 3 items were removed to create the 30 items in the pilot CD-FAB to be used in study two.

## 5. Study 2: Item Analysis and Exploratory Factor Analysis

Study two identified items for the final scale. The pilot CD-FAB was distributed to a new sample of people with CD recruited from our research database (November–December 2015). This database consists of 157 adults (18–69 years) with self-reported biopsy confirmed diagnosis of CD, recruited from online forums, who had previously volunteered to take part in research. As in study 1, individuals reporting CD diagnosis via blood test, physician report, or self-report, without intestinal biopsy, were excluded from the analyses. All 157 individuals were approached and directed to an online questionnaire, which included demographic information (age and years with diagnosis) and the pilot CD-FAB.

### 5.1. Data Analysis

Only items that contributed to the questionnaire's explanatory power were retained (see Table 2 for removal criteria) [15]. One theoretically relevant item was retained due to its salience during the focus group, “*I am afraid to touch gluten-containing foods*,” despite the removal criteria.

Principle component analysis with orthogonal rotation was used to identify loading patterns within the CD-FAB. The scree plot and factor eigenvalues > 1 identified the most appropriate factor solution.

## 6. Results of Item Analysis and Exploratory Factor Analysis

One hundred and two individuals (96 females) completed the pilot stage (mean age = 8.6 years; SD = 16.73; 9.6 years with CD diagnosis; SD = 18.24; response rate = 65%). Twelve were excluded, as they did not report a biopsy-confirmed diagnosis of CD.

### 6.1. Content and Internal Reliability

The CD-FAB was reduced from 30 to 13 items based on the criteria described in Table 1. The Cronbach's alpha for the overall scale was acceptable (0.89).

### 6.2. Exploratory Factor Analysis

The Kaiser-Meyer-Olkin and Bartlett's test of sphericity assumptions were met. A three-factor solution was extracted. Given the high intercorrelations among the three factors, a stricter eigenvalue of 2 was used, producing a one-factor solution explaining 44.1% of the variance (Table 3).

## 7. Study 3: Confirmatory Factor Analysis, Psychometrics, and Psychosocial Correlates

Study three assessed the feasibility, reliability, and psychometric properties of the CD-FAB and validated the underlying factor structure. The psychosocial correlates of the CD-FAB were also explored (see Table 4 for CD-FAB).

Recruitment posters in food outlets across the University of Birmingham, UK, directed interested individuals to an online survey (January–March 2016). Individuals were asked not to complete the questionnaire if they had completed study one. All participants who completed the questionnaire (time 1) were invited to complete the CD-FAB and items assessing predictive validity four weeks later (time 2). Online questionnaires to assess the psychosocial correlates were distributed at time 2 with the CD-FAB. Two hundred individuals with self-reported, biopsy-confirmed CD were targeted, as this is a sufficient sample size for confirmatory factor analysis (CFA) [16]. The inclusion/exclusion criteria were as described in study one.

### 7.1. Data Analysis

Floor and ceiling effects determined feasibility of the CD-FAB score; these were considered when more than 15% of respondents achieved the lowest/highest possible score.

CFA was used to confirm the one factor model found in study two, based on the goodness fit and assessed using several indices: the comparative fit index (CFI; >0.95 indicates acceptable fit), Tucker-Lewis index (TLI; >0.95 indicates acceptable fit), and root mean square errors of approximation (RMSEA; <0.08 indicates acceptable fit) [17]. Modification indices determined changes made to improve model fit. CD-FAB scores were calculated by summing the responses on each item. CD-FAB scores ranged between 13 and 91, with higher scores indicating greater CD-related food concerns and compensatory behaviours.

Correlation coefficients were used to assess test-retest reliability of the CD-FAB scores; for 50 participants, a correlation coefficient > 0.7 is indicative of strong reliability [18].

Psychosocial correlates were explored by applying a tertiary split to CD-FAB scores, dividing individuals into high, medium, and low scorers based on the 33rd and 66th percentiles. Analysis of variance was used to compare psychosocial outcomes across the three groups, and *t*-tests were used to compare the means across the low and high scorers on the CD-FAB.

### 7.2. Measures

#### 7.2.1. Food Neophobia Scale (FNS) [19]

Convergent validity was assessed using the FNS, as this has previously been used to assess food anxieties in CD^6^. The FNS measures willingness to try new food, with lower scores indicating a greater willingness to try new foods. The scale consists of 10 items and is the standard measure of food nephobia [19]. Correlations were sought to determine the degree to which the CD-FAB score reflected a fear of trying new foods. We anticipated a moderately positive relationship between total CD-FAB score and FNS scores, as individuals with high CD-FAB scores may also be fearful of trying new foods.

#### 7.2.2. Depression, Anxiety, Stress Scale 21 (DASS-21) [20]

The *Anxiety* subscale from the DASS-21 was used to assess convergent validity. This subscale measures behavioural feelings of anxiety over the last four weeks with higher scores indicating greater anxiety. The DASS-21 has strong psychometric properties; with higher scores indicating greater anxiety [21]. To demonstrate convergent validity, total scores on the CD-FAB should correlate with scores on the *Anxiety* subscale. At time 2, all subscales of the DASS-21 were distributed to explore the relationship between CD-FAB scores, depression, anxiety, and stress.

#### 7.2.3. CD Quality of Life Scale (CD-QoL) [22]

The *Treatment* subscale was used to assess discriminative validity. This subscale assesses satisfaction with one's treatment (the GFD). No relationship between these scores was anticipated. At time 2, all subscales of the CD-QoL were distributed to explore the relationship between CD-FAB scores and quality of life.

#### 7.2.4. Behavioural Item

Known groups discriminant validity was assessed using the behavioural item, *“Do you consider yourself to be anxious around food?”* This item was rated yes/no. A further behavioural item “*How many times have you eaten outside the home over the last month?”* was assessed at time 2, to assess predictive validity. We anticipated that individuals scoring high on the total CD-FAB score at time 1 would eat outside the home less than those with lower CD-FAB scores at time 2.

#### 7.2.5. Gluten-Free Management

Gluten-free dietary management was rated on a 5-point Likert scale, in response to the question “*In general, how strictly do you maintain a gluten-free diet?*” *ranging from ‘1) All of the time'; 2) ‘Most of the time'; 3) ‘Some of the time'; 4) ‘Now and then'; 5) ‘Not at all'* [23].

## 8. Results of Confirmatory Factor Analysis and Psychometrics

### 8.1. Participants at Time 1

203 (35 males, 2 “other”) participants took part in the validation stage with a mean age of 30.9 years (SD = 11.4) and 6.2 years with CD diagnosis (SD = 8.4). Nineteen participants were excluded as they reported a self-diagnosis and not a biopsy-proven diagnosis of CD. This sample was older than the participants recruited for study two (t (1, 285) = −4.21, *p* < 0.001). No difference was found in years since the diagnosis across the two samples (t (1, 286) = −1.81, *p* = 0.072). 56.1% of participants with CD reported following their GFD “*all of the time*.” Of the remainder, 1% were completely nonadherent, and 42.9% were partially adherent to the GFD.

### 8.2. Participants at Time 2

*112* of those recruited at time 1 consented to be contacted at time 2; of these, 67 completed the second questionnaire (54 females; mean age = 32.8, SD = 16.51; mean years with diagnosis = 7.5, SD = 11.42; response rate = 60%).

When comparing participant contact details to those recruited in study one, there was a 3% overlap across the samples. These individuals were removed from the analysis.

#### 8.2.1. Reliability and Feasibility

Floor and ceiling effects ranged between 0.5 and 1%. The CD-FAB total score showed good internal consistency (Cronbach's alpha = 0.89).

#### 8.2.2. Confirmatory Factor Analysis

**Figure 2:**
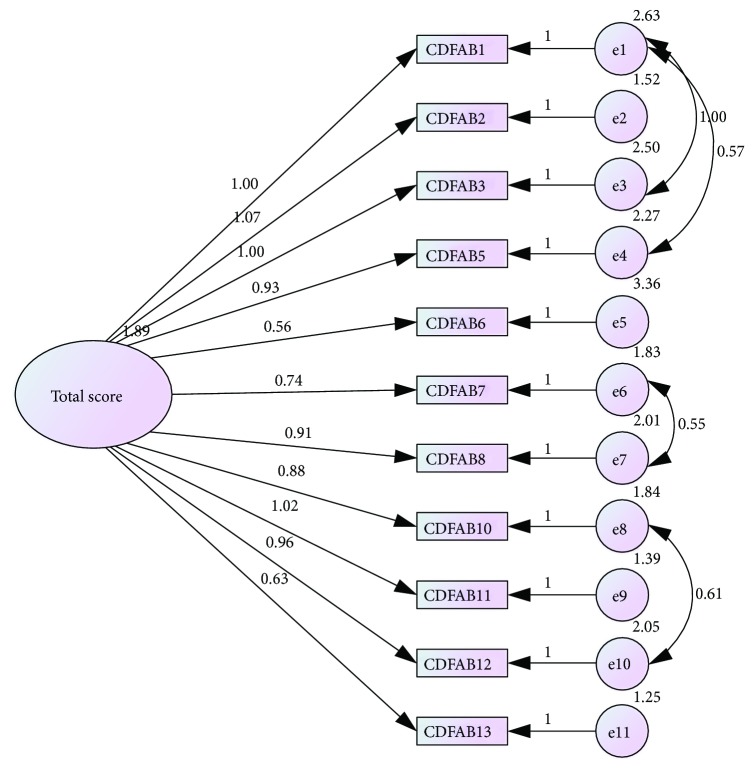
Confirmatory factor analysis with standardised item loadings onto the one CD-FAB factor. The numbers shown on the diagram from right to left are (1) covariance of the errors, (2) error terms (E), and (3) path coefficients of indicators.

All items loaded onto the total CD-FAB score (Figure 2). Figure 2 shows the structural equation model containing the standardised path estimates between the items and factors for the final model. Items 4 (*I have a lack of variety in my diet*) and 9 (*I will happily prepare gluten for others*) were removed from the model due to low factor loadings and improved model fit after removal. Despite item 6 (*I find it hard to eat gluten-free foods that look like the gluten-containing-foods that have made me ill in the past*) having a low factor loading (0.56), this item was retained, as removal did not improve model fit. Model fit could be improved by covarying the errors on items 1 and 3, 1 and 5, 7 and 8, and 10 and 12. The resulting model fit was acceptable (TLI = 0.95; CFI = 0.93; RMSEA = 0.08). The resulting CD-FAB contained 11 items with total scores ranging from 11 to 77 (Cronbach's alpha for 11-item CD-FAB remained at 0.89). These calculations were used in subsequent analyses.

### 8.3. Convergent Validity

Total CD-FAB positively correlated with the FNS (*r* = .274, *p* < 0.001) and the *Anxiety* subscale of the DASS-21 (*r* = 0.188, *p* = 0.016). Correlation coefficients were <0.7, indicating a weak relationship.

### 8.4. Discriminant Validity

Beliefs about the effectiveness of the GFD were not related to CD-FAB scores (*r* = −0.002, *p* = 0.98), indicating good discriminant validity.

### 8.5. Known Groups Validity

37.2% of participants considered themselves to feel anxious around food. These individuals had significantly higher CD-FAB scores (*m* = 48.2, SD = 6.3) than those who were not anxious around food (*m* = 40.6, SD = 7.5, *p* < 0.001)

#### 8.5.1. Predictive Validity

CD-FAB scores taken at time 1 were associated with responses to the item “*How many times have you eaten outside the home over the last month?”* taken at time 2 (*r* = −0.37, *p* = 0.048),

#### 8.5.2. Test-Retest Reliability

Test-retest correlation coefficients between the total CD-FAB scores at time 1 and time 2 were strong (*r* = 0.92, *p* < 0.001).

#### 8.5.3. Psychosocial Correlates

The top third of CD-FAB scores were associated with increased psychological distress and a more impaired quality of life when compared to low scores (*p* < 0.01). No significant differences were found between medium and high scorers (Table 5).

Low CD-FAB scorers were in the “normal” ranges for DASS-21, whereas the medium and high scorers were within the “mild” and “moderate” ranges [20]. No differences were found across age and BMI for CD-FAB scores.

## 9. General Discussion

The recent research has highlighted the association between disordered eating and CD [5–9]. Qualitative studies suggest that existing measures of disordered eating do not identify all atypical eating patterns reported in CD [13]. Beliefs around cross-contamination and food safety have been implicated in the development of disordered eating attitudes and behaviours in CD [13], but there are no tools to measure these factors. We developed and validated a self-report food attitudes and behaviours measure for adults with CD.

The CD-FAB set out to measure the four themes identified in focus groups, which explored underlying food attitudes, concerns, and eating behaviour themes (i.e., *handling of food*, *trust*, *risk-taking*, *and food safety*). One factor emerged but items targeting each of the themes were retained within this factor. This factor explained 44.1% of the variance in scores. High scores described greater concerns around food alongside avoidance and changes in the diet to cope with these concerns.

The CD-FAB has a high Cronbach's alpha (0.89), indicating strong psychometric properties and good predictive validity. Test-retest reliability over 4 weeks was excellent. This may indicate that CD food attitudes and behaviours are a stable trait, supporting previous literature highlighting this issue [24]. Additionally, the CD-FAB has good discriminant validity, showing no correlation with the CD-QoL *Treatment* subscale. The direction and magnitude of the correlations between the CD-FAB and the FNS and *Anxiety* subscale of the DASS-21 were weak, indicating limited convergent validity. However, the use of the DASS-21 to assess trait anxiety may be problematic if the CD-FAB assessed situational concerns, future research should explore the use of a state measure of anxiety alongside the CD-FAB to further explore convergent validity claims of the CD-FAB. Individuals with high CD-FAB scores felt more socially limited and concerned about their CD and its health consequences compared to low scorers, suggesting high scores on the CD-FAB indicate impaired psychosocial well-being.

A strength of this study lies in the use of participant involvement to create the CD-FAB. This information alongside a priori themes, including a framework developed by Satherley, Howard, and Higgs [2], was used to make these items relevant to participant experiences. Constructs identified by respondents related to social settings, gastrointestinal symptoms, and eating behaviours are measured for the first time by the CD-FAB. Pertinent examples of this are that eating at social events is less enjoyable after a CD diagnosis as the GFD can lead to feelings of embarrassment, isolation, and a fear of gastrointestinal symptoms, [24] for some, this fear of symptoms and anxiety around food may lead to disordered eating attitudes and behaviours [13].

Despite the strengths of the current study, future research needs to examine long-term changes in CD-FAB scores, particularly during the first year of diagnosis, to explore typical and atypical adaptation to CD and the GFD. The high dropout rate between time 1 and time 2 completion of the CD-FAB is a further limitation of this study. Given this high dropout, future investigations should consider recruiting a larger subject population for follow-up analyses. Furthermore, participants in the developmental stages of the CD-FAB were predominantly female, which reflects the higher prevalence of both eating concerns and CD in females [25, 26]. However, further validation of the CD-FAB should reflect a better balance between males and females. Additionally, despite the exclusion of participants who did not report a biopsy confirmation of their CD diagnosis throughout the procedures, self-report is not sufficient to ensure CD diagnosis. Given the confusion among CD diagnosis and symptomatic overlap with noncoeliac gluten sensitivity and irritable bowel syndrome, it is possible that individuals without a biopsy-confirmed diagnosis of CD could have been included in these studies. Given this limitation, further validation is needed in a sample of biopsy-confirmed individuals with CD recruited from clinical settings.

Measurement of symptom severity needs to be conducted alongside further assessment of the CD-FAB to control for impact of symptom severity on associated behaviours, affect, and cognition. In addition, a more thorough assessment of GFD adherence and an exploration of eating disorder history would aid in further understanding scores on the CD-FAB. Finally, expert review was not used to assess content validity, but the involvement of service users with CD in generating the items and providing feedback on the overall CD-FAB provides evidence for content validity.

These limitations do not detract from the clinical utility of the CD-FAB. The instrument may be used as an outcome measure in clinical research, enabling a greater understanding of CD-related eating patterns that are not currently captured by current tools. There is a need to establish clinical cut-off points; further guidance regarding the interpretation of CD-FAB scores (e.g., referral to dietician, clinical psychologist, or specialist eating disorder service) can be given following the identification of population norms and health implications.

The CD-FAB is a brief, self-report questionnaire that shows good reliability and validity in measuring disordered eating attitudes and behaviours in CD. The measure may be a useful tool to help understand eating attitudes and behaviours in adults with CD.

## Figures and Tables

**Table 1:** Participant characteristics for focus groups (study 1: item generation).

Pseudonym	Gender	Age (years)	Years since diagnosis
Alex	Male	36	1
Ari	Female	23	4
Ashley	Female	19	2
Charlie	Female	26	3
Clem	Female	47	2
Eddie	Female	28	3
Frances	Female	24	13
Jamie	Female	31	12
Sam	Female	29	4
Sean	Female	27	7
Tyler	Male	20	9
Wren	Female	39	14

**Table 2:** Removal criteria for CD-FAB items.

Spread of responses across options	High endorsement of a single item suggests poor discriminatory power. Items were considered for removal if >80% or <20% were an agree-type statement or a disagree-type statement	16 removed

Internal consistency	Items with a corrected item-domain total correlation < 0.3 or in a domain with a poor Cronbach's alpha < 0.7 were considered for removal	1 removed

Timing of administration of questionnaire	Needs to be applicable to people from the point of coeliac disease diagnosis onwards, so all individuals with coeliac disease can complete the scale	2 reworded

Clarity and relevance of items	Difficult to understand items were reworded or considered for removal	13 reworded

Items deemed theoretically important	These items were retained despite meeting the above criteria because they were deemed theoretically important	1 retained

**Table 3:** Factor loadings for CD-FAB items.

	Item number	Factor loading
Cronbach's alpha for scale		
I am afraid to eat outside my home	2	0.78
I am comfortable eating gluten-free food from other people's kitchens^∗^	11	0.77
I am afraid to touch gluten-containing foods	3	0.75
I get concerned being near others when they are eating gluten	1	0.73
My concerns about cross-contamination prevent me from going to social events involving food	8	0.71
I get worried when eating with strangers	5	0.70
I enjoy going out for meals as much as I did before my diagnosis^∗^	10	0.69
Being contaminated by gluten in the past has not stopped me from enjoying restaurants^∗^	12	0.69
I will only eat food that I have prepared myself	7	0.65
If I ask questions, I can normally find gluten-free food to eat^∗^	13	0.64
I will happily prepare gluten for others	9	0.58
I find it hard to eat gluten-free foods that look like the gluten-containing foods that have made me ill in the past	6	0.45
I have a lack of variety in my diet	4	0.35

^∗^ represents items that are reverse scored. Numerical values represent factor loadings.

**Table 4:** The Coeliac Disease Food Attitudes and Behaviours scale (CD-FAB). Instructions: this questionnaire is designed to explore food attitudes and beliefs in coeliac disease. Some questions may not apply to you; this is because we are trying to assess a range of beliefs about coeliac disease and managing the gluten-free diet. Please fill out the form below as accurately, honestly, and completely as possible. There are no right or wrong answers. All of your responses are confidential. Please tick the box that best describes your response to the question.

	Strongly agree (7)	Agree (6)	Somewhat agree (5)	Neither agree nor disagree (4)	Somewhat disagree (3)	Disagree (2)	Strongly disagree (1)
*Because of my coeliac disease…*							
I get concerned being near others when they are eating gluten							
I am afraid to eat outside my home							
I am afraid to touch gluten-containing foods							
I get worried when eating with strangers							
I find it hard to eat gluten-free foods that look like the gluten-containing foods that have made me ill in the past							
I will only eat food that I have prepared myself							
My concerns about cross-contamination prevent me from going to social events involving food							
*Despite having coeliac disease…*							
I enjoy going out for meals as much as I did before my diagnosis^∗^							
I am comfortable eating gluten-free food from other people's kitchens^∗^							
Being contaminated by gluten in the past has not stopped me from enjoying restaurants^∗^							
If I ask questions, I can normally find gluten-free food to eat^∗^							

Reverse items with ^∗^ and add all scores to make total score.

**Table 5:** Demographic and psychosocial outcomes using the tertiary split on the CD-FAB. Data are presented as means.

	Low scorers	Medium scorers	High scorers	F statistic
*Demographic outcomes*				
Age (years)	32.3	29.0	29.0	1.75
BMI	22.6	22.7	21.9	0.29
*Psychosocial outcomes*				
Depression	8.6	13.5^a^	13.5^b^	3.81^∗^
Anxiety	6.6	10.1^a^	11.6^b^	4.34^∗^
Stress	11.6	16.9^a^	15.8^b^	4.13^∗^
Total DASS-21	26.7	40.5^a^	40.9^b^	5.3^∗^
FNS	27.4	31.3	33.9^b^	10.7^∗∗^
Total quality of life	71.1	57.9^aa^	53.6^bb^	16.03^∗∗^
Limitations	31.4	24.4^aa^	21.9^bb^	17.32^∗∗^
Health	17.1	13.6^aa^	12.4^bb^	10.92^∗∗^
Treatment	5.5	5.5	5.5	0.032
Dysphoria	17.1	14.6^a^	14.2^bb^	7.12^∗∗^

^∗^ *p* = 0.05; ^∗∗^*p* < 0.001 for ANOVA across all three groups; ^a^*p* = 0.05; ^aa^*p* < 0.001 for *t*-test across low and medium scorers; ^b^*p* = 0.05; ^bb^*p* < 0.001 for *t*-test across low and high CD-FAB scorers.

## Data Availability

The data used to support the findings of this study are available from the corresponding author upon request.

## Abbreviations

BES: Binge eating scale
CD: Coeliac disease
CD-FAB: Coeliac Disease Food Attitudes and Behaviours scale
CD-QoL: Coeliac disease quality of life scale
CFI: Comparative fit index
DASS-21: Depression anxiety stress scale
EAT-26: Eating attitudes test
FNS: Food Neophobia Scale
GFD: Gluten-free diet
RMSEA: Root mean square of errors approximation
TLI: Tucker-Lewis index.
